# Pharmacological Characterization of the Novel CRF1 Receptor Antagonist, Thiazolo[4,5-d] Pyrimidine Analog, M43

**DOI:** 10.3390/biom15091265

**Published:** 2025-09-01

**Authors:** Spyridon Marios Giatro, George Komontachakis, Aikaterini Kalantidou, Nastazia Lesgidou, Vlasios Karageorgos, Mohamed Teleb, Md Rabiul Islam, Thomas Mavromoustakos, Hesham Fahmy, Maria Venihaki, Minos-Timotheos Matsoukas, George Liapakis

**Affiliations:** 1 Department of Biomedical Engineering, University of West Attica, 12210 Egaleo, Greece; sgiatro@uniwa.gr (S.M.G.); nlesgidou@uniwa.gr (N.L.); 2 Department of Pharmacology, School of Medicine, University of Crete, 71003 Heraklion, Greece; geokomontas@gmail.com (G.K.); bkarageorgos@hotmail.com (V.K.); 3 Department of Clinical Chemistry, School of Medicine, University of Crete, 71003 Heraklion, Greece; katekld7@gmail.com (A.K.); venycham@uoc.gr (M.V.); 4 Department of Pharmaceutical Chemistry, Faculty of Pharmacy, Alexandria University, Alexandria 21521, Egypt; mohamed.t.ismail@alexu.edu.eg; 5 Department of Medicinal Chemistry, Faculty of Pharmacy, Alamein International University, Alamein 51718, Egypt; 6 Department of Pharmaceutical Science, College of Pharmacy & Allied Health Professions, South Dakota State University, Brookings, SD 57007, USA; mdrabiul.islam925@jacks.sdstate.edu (M.R.I.); hesham.fahmy@sdstate.edu (H.F.); 7 Department of Chemistry, National and Kapodistrian University of Athens, 15771 Athens, Greece; tmavrom@chem.uoa.gr

**Keywords:** non-peptide antagonists, corticotropin-releasing factor, receptor, signaling, structure

## Abstract

The corticotropin-releasing factor (CRF) and its type 1 receptor (CRF_1_R) play a key role in the regulation of the hypothalamic–pituitary–adrenal (HPA) axis. Dysregulation of the HPA axis is associated with congenital adrenal hyperplasia (CAH) and depression. Non-peptide CRF_1_R-selective antagonists displayed antidepressant effects on animal models and are used for the management of CAH. To develop novel non-peptide CRF_1_R antagonists, we have previously designed and synthesized a series of substituted pyrimidines. Among these analogs, molecule 43 (M43) binds to CRF_1_R with the highest affinity. Based on this finding, we selected M43 for further pharmacological characterization in the present study. The results suggest that M43 is a potent CRF_1_R antagonist, blocking the ability of the CRF-related agonist, Tyr^0^-sauvagine, to stimulate (1) cAMP accumulation in HEK 293 cells expressing CRF_1_R and (2) the proliferation rate of RAW 264.7 macrophages. Computational studies suggest that the antagonist properties of M43 are mostly attributed to its ability to interact with residues in the allosteric pocket of CRF_1_R, comprised of the third, fifth, and sixth transmembrane domain residues, which block activation-associated structural rearrangements of the receptor. Our data will be used to design novel non-peptide CRF_1_R antagonists for clinical use.

## 1. Introduction

The hypothalamic corticotropin-releasing factor (CRF or CRH) is a 41-amino acid peptide that exerts multiple biological actions through its interaction with the type 1 (CRF_1_R). The CRF receptors belong to family B of G-protein-coupled receptors (GPCRs) [1,2]. Activation of CRF receptors by the CRF and related agonists, including Tyr^0^-sauvagine, leads to a biological effect by stimulating, through the Gs-proteins, the intracellular cAMP accumulation [1,2].

CRF and its receptors are essential for the maintenance of homeostasis because they regulate the hypothalamic–pituitary–adrenal axis (HPA) [3]. Specifically, CRF is secreted from the hypothalamus and stimulates the secretion of corticotropin (ACTH) from the pituitary through its interaction with the CRF_1_R [2,4]. The ACTH subsequently stimulates the release of glucocorticoids from the adrenals [2,3]. The HPA axis undergoes negative feedback regulation by the glucocorticoids, which suppress the release of CRF and ACTH [5]. In addition to the regulation of the HPA axis, the CRF and CRF_1_R play an important role in the function of the central nervous system (CNS) [6,7,8,9].

Hyperactivity of the HPA axis and dysregulation of the CRF/CRF_1_R circuits of the CNS caused by various factors, such as chronic stress, are associated with maladaptation to stress and the appearance of stress-related disorders, such as anxiety and depression [5,7,10,11,12,13,14]. Injection of CRF in the CNS of model animals or overexpression of CRF in transgenic mice is associated with anxiety and depressive-like symptoms [15,16,17,18,19]. These actions of CRF in the CNS are mostly mediated through its interaction with the CRF_1_R, as suggested in previous studies involving CRF_1_R antisense oligonucleotides or CRF_1_R-deficient animals [9,20]. Accordingly, non-peptide CRF_1_R antagonists appeared to have antidepressant and anxiolytic properties in experimental animals, with several of them entering clinical trials [21,22].

Dysregulation of the HPA axis is also observed in several pathophysiological conditions, such as congenital adrenal hyperplasia (CAH), which is characterized by decreased glucocorticoid secretion from the adrenals and impaired glucocorticoid feedback inhibition of the HPA axis [23,24]. This results in augmented secretion of CRF and ACTH, leading to the excessive production of adrenal androgens. The CAH is an autosomal recessive disorder caused by 21-hydroxylase deficiency [23,24]. The severity of CAH is closely associated with the degree of 21-hydroxylase deficiency, with the most severe type being the classic salt-wasting one, which is characterized by mineralocorticoid and cortisol deficiency that leads to salt loss and varying degrees of prenatal virilization of external genitalia [23,24]. Kyritsi et al. have proposed that CAH carriers could be more prone to the development of anxiety disorders, in agreement with their findings, which indicated a chronic hyperactivity of the HPA axis due to its impaired feedback inhibition by glucocorticoids [25]. Similarly, Harasymiw et al. have shown that children, adolescents, and young adults with CAH were more likely to be diagnosed with a depressive or anxiety disorder compared to their healthy counterparts [26]. Treatment of CAH aims to replace cortisol and reduce the excess of androgens [23,24]. Administration of cortisol restores its decreased levels and decreases the excessive production of adrenal androgens by re-establishing the negative feedback of the HPA axis [23,24,27]. However, glucocorticoid replacement therapy often requires supraphysiological doses to suppress androgen excess, thus putting CAH patients at the risk of glucocorticoid overexposure, leading to conditions such as growth suppression, metabolic disorders, insulin resistance, and hypertension [23,24,27,28]. An approach to reducing the overproduction of adrenal androgens while administering glucocorticoids at physiological doses is to decrease the ACTH secretion by administering CRF_1_R antagonists [27,28,29,30]. Based on this concept, two CRF_1_R non-peptide antagonists, tildacerfont and crinecerfont, have been developed and tested for the management of CAH [29,31].

The development of tildacerfont for the treatment of CAH was terminated because it failed to meet its primary endpoint of glucocorticoid dose reduction in a clinical phase 2b trial [29]. Similarly, although several CRF_1_R non-peptide antagonists are currently in clinical trials for the treatment of depression and anxiety, none is in clinical use [21]. In contrast, crinecerfont, after successfully passing phases 2 and 3 of clinical trials, was approved in December 2024 for the treatment of pediatric and adult patients with classic CAH [28,29,31]. However, despite its high tolerability, crinecerfont has several potentially severe side effects, although less common, including hypersensitivity reactions and suicidal ideation and behaviors [32].

The drawbacks of using CRF_1_R non-peptide antagonists as drugs for the treatment of CAH and stress-related disorders highlight the need for the development of novel molecules with better pharmacological and safety profiles. In an effort to develop novel CRF_1_R antagonists, we have designed, synthesized, and tested the binding properties of 45 non-peptide molecules [33]. Among these compounds, the analog 43 (or M43) has been shown to bind to CRF_1_R with the highest affinity. In the present study, we rationalized the relationship between M43/CRF_1_R interactions and the binding affinity of M43 by constructing several molecular models and experimentally determined the antagonistic properties of M43.

## 2. Materials and Methods

### 2.1. Cell Culture

Human embryonic kidney (HEK) 293 cells stably expressing the human CRF1R were generated as previously described [34] and were grown in Dulbecco’s modified Eagle’s medium (DMEM)/F-12 (1:1) containing 3.15 g/L of glucose and 10% bovine calf serum at 37 °C and 5% CO_2_. One day before the experiment, the cells were plated in 96-well cell culture plates (pretreated with 0.1 mg/mL poly-L-lysine) at a density that resulted in 95–100% confluency within one day.

Mouse macrophage cell line RAW 264.7 was cultured using DMEM-high glucose medium (Gibco, Jenks, OK, USA, 41966-029) supplemented with 10% heat-inactivated Fetal Bovine Serum (Gibco, 10270-106) and 1% penicillin streptomycin (Gibco, 15140-122) at 37 °C in the presence of 5% CO_2_. Macrophage activation was induced using 100 ng/mL LPS (L2630, Sigma, St. Louis, MO, USA). For proliferation assays, RAW 264.7 cells were plated in 96-well plates at a concentration of 4.000 cells/well for 24 h. Then, cells were treated with analog M43 at 1 μM and/or Tyr^0^-Sauvagine at 10 nM for 72 h.

### 2.2. cAMP Accumulation Assays

HEK 293 cells stably expressing CRF_1_R were initially incubated in 100 μL of assay buffer at 37 °C. After 1 h, additional buffer—with or without ligands (to assess basal activity)—was added to a final volume of 200 μL, and incubation continued for another 30 min at 37 °C. Varying concentrations of Tyr^0^-sauvagine were applied, both in the presence and absence of 1 μM analog M43, to assess the ligand’s potency. Parallel assays were conducted with a fixed 10 nM concentration of Tyr^0^-sauvagine, with increasing concentrations of analog M43 to determine its half-maximal inhibitory concentration (IC_50_). Following incubation, the buffer was removed, and the cells were placed on ice and lysed using 3% trichloroacetic acid (TCA). Lysates were kept on ice for 30–60 min and then stored at −20 °C. After storage for 1–5 days, samples were thawed and centrifuged at 1800× *g* for 10 min at 4 °C, and supernatants were neutralized using 2 N NaOH. Intracellular cAMP levels were quantified via a competitive binding assay. In brief, 20 μL of each neutralized sample was transferred to 3 mL polypropylene tubes containing buffer A (100 mM Tris-HCl, pH 7.4, 100 mM NaCl, and 5 mM EDTA) and 1 nM of [2,8-^3^H] cAMP. Approximately 100 mg of bovine adrenal cortex extract (serving as the cAMP-binding protein) diluted in 500 mL of buffer A was then added. After 3 h of incubation on ice, samples were filtered through Whatman 934AH glass fiber filters using cold buffer C (120 mM NaCl and 10 mM Tris-HCl, pH 7.4) as the wash solution and a Brandel filtering device.

The amount of cAMP was measured against a logarithmic standard curve (1–100 pmol/tube) using known concentrations of unlabeled cAMP. IC_50_ values for analog M43 (expressed as pIC_50_) were derived using a one-site competition model via nonlinear regression (Prism 8.0). The IC_50_ represents the concentration of analog M43 needed to inhibit 50% of cAMP production induced by 10 nM sauvagine. Potency values for Tyr^0^-sauvagine (expressed as −LogEC_50_), in the presence and absence of analog M43, were determined using a one-site sigmoidal fit model.

### 2.3. Proliferation Assays

Cell proliferation in RAW 264.7 macrophages was assessed following treatment with 10 nM of sauvagine, 1 nM of analog M43, or their combination, using the MTT assay (Sigma). Cells were seeded into flat-bottomed 96-well plates and, after 24 h, were incubated with 100 ng/mL of LPS (L2630, Sigma), analog M43 at 1 μM, and/or Tyr^0^-sauvagine at 10 nM at 37 °C in the presence of 5% CO_2_. After 72 h, MTT (3-(4,5-dimethylthiazol-2-yl)-2,5-diphenyltetrazolium bromide) was added to each well at a final concentration of 0.5 mg/mL, and cells were incubated for 4 h at 37 °C. Mitochondrial dehydrogenases in viable cells converted MTT into insoluble formazan crystals. These were subsequently dissolved in DMSO, and absorbance was measured at 595 nm using a Dynatech MicroElisa plate reader (Chantilly, VA, USA).

### 2.4. Model Generation of Receptor–Ligand Complexes

We utilized AlphaFold3 to model the binding of ligands to the CRF_1_R [35]. The sequence used to generate the model was derived from UniProt (P34998) (post-translational modification sequence ID: PRO_0000012814), encompassing amino acids 24-415. We generated the apo receptor model using the abovementioned sequence (protein only) and complex models (protein with each of the nine compounds). Compounds were added to the JSON input file as SMILES strings. Additionally, 50 phospholipids (ccd code: HWP) were added to each model to better simulate the lipid bilayer environment. The generated models were compared with known crystallographic CRF_1_R structures, specifically 8GTI (inhibitor-bound) and 6P9X (activated complex) [36,37]. We assessed model confidence and relative positioning using AlphaFold’s provided metrics: the predicted local distance difference test (pLDDT) for local confidence and the predicted alignment error (PAE) for global confidence and relative positioning.

### 2.5. Molecular Dynamics Simulations

Molecular dynamics simulations of the CRF_1_R in complex with the small-molecule antagonist M43 were performed using GROMACS 2023.4 [38]. The initial structure used was the AlphaFold-predicted model of the M43–CRF_1_R complex. Parameterization of the protein–ligand complex was performed using CHARMM-GUI [39]. The ligand topology was generated using the CHARMM General Force Field (CGenFF), and the protein was embedded into a pre-equilibrated 1-palmitoyl-2-oleoyl-sn-glycero-3-phosphocholine (POPC) lipid bilayer of dimensions 120 Å × 120 Å, resulting in a membrane patch containing 387 POPC molecules [40]. The system was solvated with TIP3P water and neutralized with Na^+^ and Cl^−^ ions to achieve an approximate ionic strength of 0.15 M. All simulations were conducted using the CHARMM36 force field [41]. The system was energy-minimized using the steepest descent algorithm. This was performed under positional restraints on the protein backbone, side chains, and lipid head groups with force constants of 4000, 2000, and 1000 kJ·mol^−1^·nm^−2^, respectively. Additionally, dihedral restraints were applied to backbone φ/ψ angles (force constant: 1000 kJ·mol^−1^·rad^−2^). The minimization continued until the maximum force on any atom was below 1000 kJ·mol^−1^·nm^−1^, or for a maximum of 5000 steps. A six-step equilibration scheme was employed to gradually relax the system toward physiologically relevant dynamics while preserving the overall structural integrity of the receptor–ligand complex: first, 1.5 ns of NVT simulation at 310 K with strong positional and dihedral restraints on protein and lipids (randomized velocities) to allow solvent and lipid tails to settle; next, 1.5 ns NVT with all restraints halved to increase solvent and side-chain mobility; then, 1.5 ns NPT with semi-isotropic C-rescale barostat (1 bar, τ = 5 ps, κ = 4.5 × 10^−5^ bar^−1^) and further reduced restraints to equilibrate the membrane; a 5 ns NPT run (2 fs timestep) with backbone restraints lowered to 500 kJ·mol^−1^·nm^−2^ for initial protein flexibility; a 10 ns NPT interval with minimal restraints (backbone 200, side chains 50, lipids 40 kJ·mol^−1^·nm^−2^) to approach near-native sampling; and finally, another 10 ns NPT under the same light restraints to fully stabilize the system before production. Throughout all equilibration steps, hydrogen bond constraints were maintained using the LINCS algorithm, and short-range van der Waals and Coulomb interactions were treated with a 1.2 nm cutoff, using force-switching from 1.0 to 1.2 nm. Long-range electrostatics were computed using the Particle-Mesh Ewald (PME) method. Following equilibration, a 500 ns production simulation was carried out at 310 K and 1 bar. All restraints were removed, allowing the receptor–ligand complex and lipid bilayer to evolve freely. A 2 fs integration timestep was used, with all bonds to hydrogen atoms constrained. Temperature coupling was applied separately to solute (protein + ligand), membrane, and solvent groups using the velocity-rescale thermostat. Semi-isotropic pressure coupling was maintained using the C-rescale barostat.

Following molecular dynamics (MD) simulations, root mean square deviation (RMSD) analyses were conducted using only the Cα atoms of residues embedded within the membrane. The highly flexible extracellular and intracellular regions were excluded to focus on assessing the structural stability of the membrane-embedded portion of the receptor.

## 3. Results

### 3.1. Computational Models of the Interactions Between CRF1R Non-Peptide Antagonists and CRF_1_R

**Figure 1:**
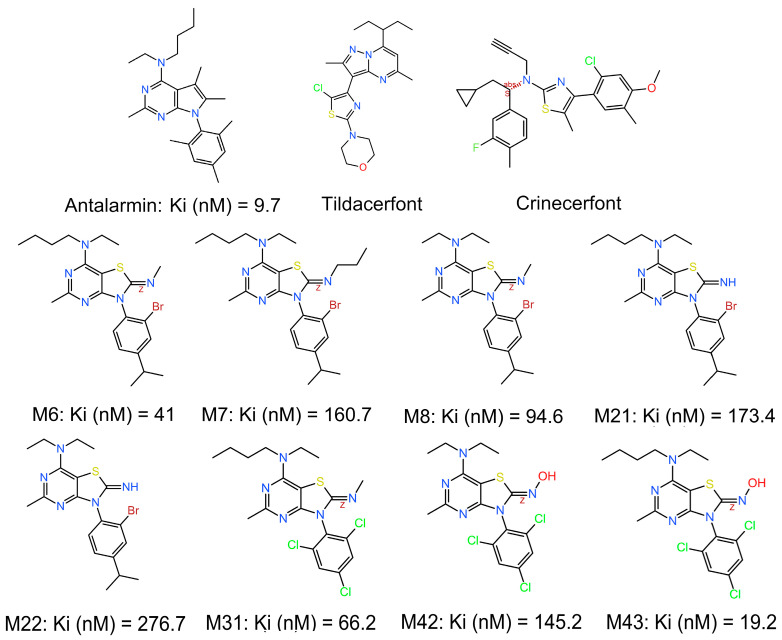
Chemical structures of eight novel non-peptide CRF antagonists and known antagonists, such as antalarmin, tildacerfont, and crinecerfontand, as well as their measured (where applicable) binding affinities for the CRF_1_R [33].

We have previously shown that out of 45 novel non-peptide molecules we designed and synthesized, eight of them bound to CRF_1_R with relatively high affinities (Figure 1) [33]. The synthesized compounds were inspired by existing CRF_1_R antagonists in the literature, but with a different scaffold (thiazolo[4,5-d] pyrimidine) in an effort to increase affinity, provide novel chemical entities, and further advance research on small molecules in the field. All the selected compounds that displayed promising activities are thiazolo[4,5]pyrimidines carrying a small alkylimino (*N*-methyl, *N*-propyl, or hydroxylamine) group at position 2, a secondary amine (diethylamine or ethyl, butylamine) at position 7, and a substituted phenyl group (2,4,6-trichlorophenyl or 2-bromo-4-isopropylphenyl) at position 3. MC43, which displayed the highest binding affinity of 19.2 nM, which is only two times lower than the prototype non-peptide antagonist antalarmin (Figure 1) [33], has a hydroxylimino functional group at position 2, a butyl, ethylamino functional group at position 7, and a 2,4,6-trichlorophenyl functional group at position 3.

**Figure 2:**
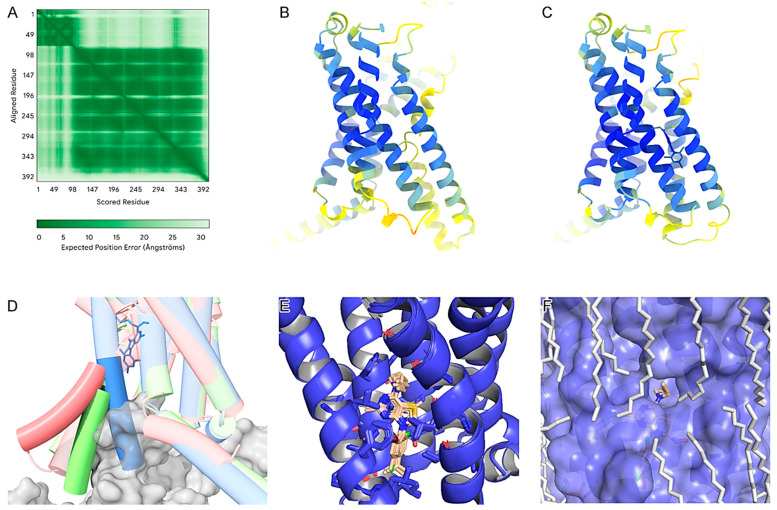
(**A**) PAE plot for the CRF_1_R ligand-free model. (**B**) Ligand-free CRF_1_R model, colored by pLDDT. (**C**) CRF_1_R in complex with M43, colored by pLDDT. (**D**) Superimposition of the M43–CRF_1_R complex (blue), CRF_1_R ligand-free model (green), and CRF_1_R active state experimental structure in a complex with CRF_1_R peptide and guanine nucleotide binding protein G(s) (pdb id: 6p9x) (red). (**E**) Superimposition of 9 ligand-protein complexes generated using AlphaFold3. (**F**) Protein–ligand–membrane complex generated using AlphaFold, highlighting a potential interface between the ligand and the membrane.

To understand the molecular determinants of the identified non-peptide ligands (M6, M7, M8, M21, M22, M31, M42, M43, and the control antalarmin) that bind to CRF_1_R, we produced AlphaFold models of the apo state and complexes with these nine compounds (Figure 2). According to AlphaFolds’ PAE (Figure 2A), the secondary structures are accurately positioned relative to each other, except for the first 80 residues forming the extracellular domain of the receptor (ECD) in relation to the rest of the protein. This indicates flexibility in the ECD. The pLDDT scores were notably high for the core amino acids of the transmembrane domain in both the apo and ligand-bound models (Figure 2B,C). All ligand-bound models exhibited higher pLDDT scores, especially within the core region of the binding pocket and its surrounding helices. This suggests that the compounds stabilize the receptor in the inactive state by interacting with the allosteric pocket between the third (TM3), fifth (TM5), and sixth (TM6) transmembrane domains, already identified by others [42]. By comparing the AlphaFold3 models, it is evident that the ligand-free model adopts an intermediate state, while the protein–ligand complex is in an inactive state. This is highlighted by an approximate 30-degree outward shift of the lower part of TM 6 (Figure 2D).

No significant local sidechain shifts around the binding site were observed among the nine compounds (Figure 2E). A primary observation across all compounds is that the thienopyrimidine group forms a hydrogen bond with N283^5.50b^, which is consistent with the previously reported literature [42]. The superscripts of receptor residues represent their positions in the TMs of the receptor, with the most conserved residue in each TM of subfamily B1 GPCRs to be assigned the position index 0.50, and this number is preceded by the TM number (TM1–TM7) [43]. Thus, N283^5.50b^ denotes that Asn283 is the most conserved residue in the TM5 of subfamily B1 GPCRs.

Furthermore, the trichlorophenyl group, which extends deep into the cavity, appears to benefit from all three halogens, as exemplified by M43. These halogens interact with hydrophobic residues (L287^5.54b^, L320^6.46b^, F284^5.51b^, I290^5.57b^) and potentially form a weak halogen bond with Y363^7.57b^. More importantly, the three chlorine atoms enhance the negative electron density on the phenyl ring, while the heavy bromine atom on M22 or M8 makes the interaction asymmetric and subsequently less stable.

Regarding the *N*-butyl-*N*-ethylamino group, consistent with K_i_ values, we propose that the addition of an even longer chain or the addition of other groups primarily on the butyl part might improve binding affinity. This is evident when comparing M43 with M42 K_i_ values, and it is supported by the AlphaFold3 model (Figure 2F), in which we observe an interface between the phospholipids and the *N*-butyl-*N*-ethylamino group of M43. This region is highly hydrophobic and positioned in the middle of the lipid bilayer, suggesting ample space for additional hydrophobic groups at this location. Potential additions in this region could include an aromatic moiety or halogens.

Finally, we observe that the oxime group of M43 and M42 forms a hydrogen bond with the carbonyl group of T316^6.42b^ backbone. This is not the case for other molecules, such as M31, which, compared to M43, lacks the hydroxyl group of the oxime, but instead has a methyl group; therefore, it does not have the ability to form such a hydrogen bond and loses affinity. Also, by substituting the oxime hydroxyl group with hydrogen (as is the case for M22), although there is a potential hydrogen donor and acceptor, the angle does not favor the formation of a hydrogen bond.

Comparing the predicted binding mode of M43 with that of antalarmin (Figure S1), it is evident that both molecules have similar binding characteristics. The thiazole of M43 has one less substituent than the pyrrolopyrimidine of antalarmin, of which the three methyl substituents increase hydrophobicity. The main difference is that M43 seems to form an extra hydrogen bond with the oxime on the thiazole group. The fact that antalarmin had a slightly higher binding affinity than M43 may be explained by the increased hydrophobicity of the core scaffold provided by its methyl groups, which is partially compensated by the extra hydrogen bond of M43 with T316^6.42b^.

### 3.2. M43 Forms Specific Hydrogen Bond Interactions with the Allosteric Pocket

**Figure 3:**
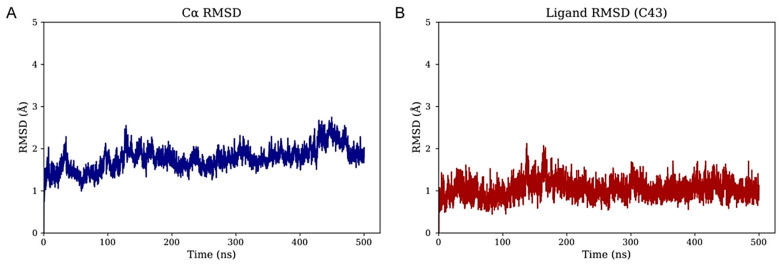
Root mean square deviation (RMSD) analysis of the protein–ligand complex. (**A**) C_α_ RMSD of the protein backbone over time, shown in blue. (**B**) Ligand RMSD after alignment on the protein C_α_ atoms, shown in red.

To investigate the structural dynamics of the CRF_1_R in complex with the top-performing small-molecule antagonist M43, unbiased molecular dynamics (MD) simulations were performed (see 1.5 Methods). The initial complex, based on the AlphaFold3-predicted model, was embedded in a POPC bilayer. The system, solvated with TIP3P water and 0.15 M NaCl, underwent energy minimization and multi-stage restrained equilibration before a 500 ns production run under physiological temperature and pressure conditions. As shown in Supplementary Figure S2, peripheral regions—particularly the extracellular domain (ECD)—exhibit pronounced conformational fluctuations and were excluded from global RMSD values. This finding is consistent with the AlphaFold3 PAE plot (Figure 2A). The resulting C_α_ RMSD remained below 3 Å throughout the simulation, indicating a stable transmembrane core (Figure 3A). Furthermore, when fitting on the C_α_ atoms of the membrane-inserted residues, the RMSD of the bound molecule M43 remained below 2 Å, demonstrating that the ligand maintains a well-defined binding pose relative to the stable membrane-embedded region of the protein (Figure 3B). These results support the structural integrity of the transmembrane domain and highlight the robustness of M43 binding during the simulation. To further validate the model and the simulation, RMSD calculations of the TMD were performed among the AlphaFold model, the most representative state from the MD simulation, and the complex of the receptor bound to BMK-C205 (pdb: 8gti) crystal structure (Figure S3A,B) [36]. BMK-C205 is an allosteric antagonist, structurally characterized in the same allosteric site of CRF_1_R. Calculations show that the TMD of the AlphaFold starting model and the representative model from the MD have an RMSD of 1.73 and 1.78, respectively, to the resolved structure. RMSD calculations of frames from the M43-CRF_1_R complex simulation, with the BMK-C205-CRF_1_R crystal structure (Figure S3C), further validate and indicate a stable dynamics simulation for the M43-CRF_1_R complex.

During the MD simulation, several hydrophobic interactions stabilize the ligand. In the co-folding AlphaFold models, the pocket is formed by L287^5.54b^, L320^6.46b^, F284^5.51b^, and I290^5.57b^, which accommodates the halogenated moiety of M43. One of the two terminal alkyl chains (M5–M6), linked via N1, is oriented toward F203^3.44b^, supporting favorable hydrophobic packing. The second alkyl chain (M1–M4) extends toward the membrane interface, positioning itself in close proximity to the surrounding POPC lipids, suggesting membrane-assisted stabilization of the ligand conformation.

**Figure 4:**
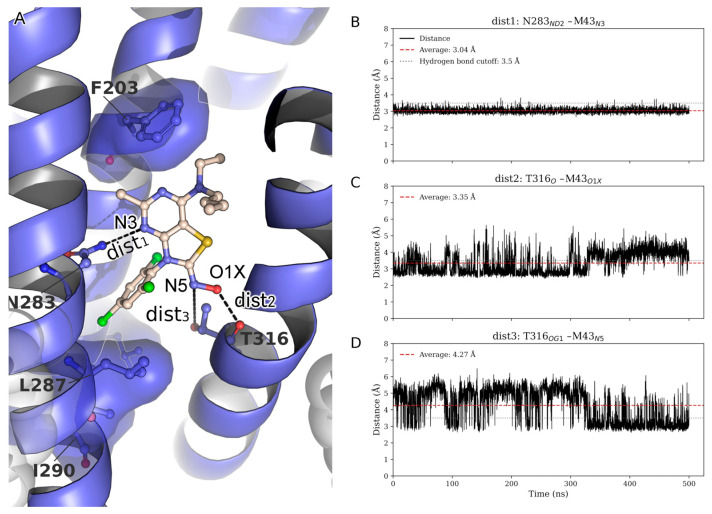
(**A**) Representative 3D structure from the simulation, showing M43 ligand (yellow) bound to CRF_1_R (blue). Dashed lines indicate the measured distances between N283^5.50b^_ND2_–M43_N3_, T316^6.42b^_O_–M43_O1X_, and T316^6.42b^_OG1_–M43_N5_, with corresponding labels. Key hydrophobic residues are highlighted with a transparent surface representation, and white spheres denote the membrane bilayer. (**B**) Time-resolved distance between N283^5.50b^_ND2_ and M43_N3_ (dist1). (**C**) Time-resolved distance between T316^6.42b^_O_ and M43_O1X_ (dist2). (**D**) Time-resolved distance between T316^6.42b^_OG1_ and M43_N5_ (dist3). Dashed red lines represent the average distance over time, while dotted gray lines indicate the 3.5 Å hydrogen bond threshold.

To further assess the persistence of key interactions stabilizing ligand M43, time-resolved distances of three putative hydrogen bonds were monitored throughout the trajectory: dist1: N283^5.50b^_ND2_–M43_N3_, dist2: T316^6.42b^_O_–M43_O1X_, and dist3: T316^6.42b^_OG1_–M43_N5_ (Figure 4A). The N283^5.50b^–M43_N3_ interaction (dist1) proved to be highly stable, as known from previous studies, consistently maintaining a distance well below the hydrogen bond threshold throughout the simulation (Figure 4A) [42]. The T316^6.42b^_O_–M43_O1X_ interaction monitored via dist2 exhibited moderate fluctuations around the cutoff value, occasionally breaking the hydrogen bond (Figure 4C). Notably, during these periods of dist2 disruption, an alternative hydrogen bond formed between T316^6.42b^_OG1_ and the N5 atom of M43 (dist3) (Figure 4D), indicating a dynamic switch between the two interactions. This complementary behavior suggests that T316^6.42b^ maintains stabilizing contact with M43 by alternating between O1X and N5, depending on local structural fluctuations. Collectively, these observations highlight a cooperative hydrogen-bonding network, with the N283^5.50b^ interaction serving as a persistent anchor and T316^6.42b^ contributing adaptable support through transient interactions with multiple sites on M43. It also indicates that dist3 is important for compounds in the series where the N5 serves as a hydrogen bond acceptor that interacts with T316^6.42b^. The fact that M31, M6, or M8 lack the hydroxyl group of the oxime, but instead have a methyl group, maintaining reasonable affinities, also indicates the important role of this hydrogen bond to the series in general.

### 3.3. M43 Antagonizes the Stimulation of cAMP Accumulation by Tyr^0^-Sauvagine

**Figure 5:**
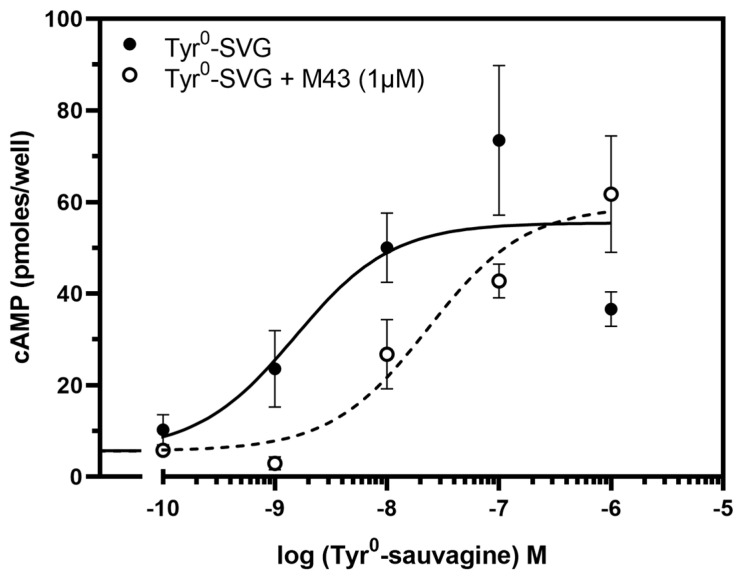
M43 decreases the potency of Tyr^0^-sauvagine to stimulate cAMP accumulation. The stimulation of cAMP accumulation in HEK293 cells stably expressing the CRF_1_R by increasing concentrations of Tyr^0^-sauvagine (Tyr^0^-SVG) in the absence or presence of 1 μM of M43 was determined as described in the “Materials and Methods”. The means and S.E. (duplicate determination) are shown from a representative experiment performed 12 times. The potencies (−logEC_50_) of SVG without or with M43, as determined from these experiments, were 8.65 ± 0.15 and 7.86 ± 0.22, respectively.

The ability of M43 to bind to CRF_1_R with the highest affinity compared to the other compounds tested in our previous study prompted us to test its antagonistic properties [33]. To accomplish this, we first determined its ability to decrease the potency of the CRF-related agonist Tyr^0^-sauvagine in stimulating cAMP accumulation in HEK 293 cells expressing the CRF_1_R. As shown in Figure 5, M43 at a concentration of 1 μΜ significantly decreased the potency of Tyr^0^-sauvagine by 6.2-fold (*p* < 0.005, paired t-test). Specifically, in the absence of M43, Tyr^0^-sauvagine’s potency was 2.2 nM (or −LogEC_50_ = 8.65 ± 0.15, n = 12), whereas in its presence, the potency of Tyr^0^-sauvagine was 13.9 nM (or −logEC_50_ = 7.86 ± 0.22, n = 12).

**Figure 6:**
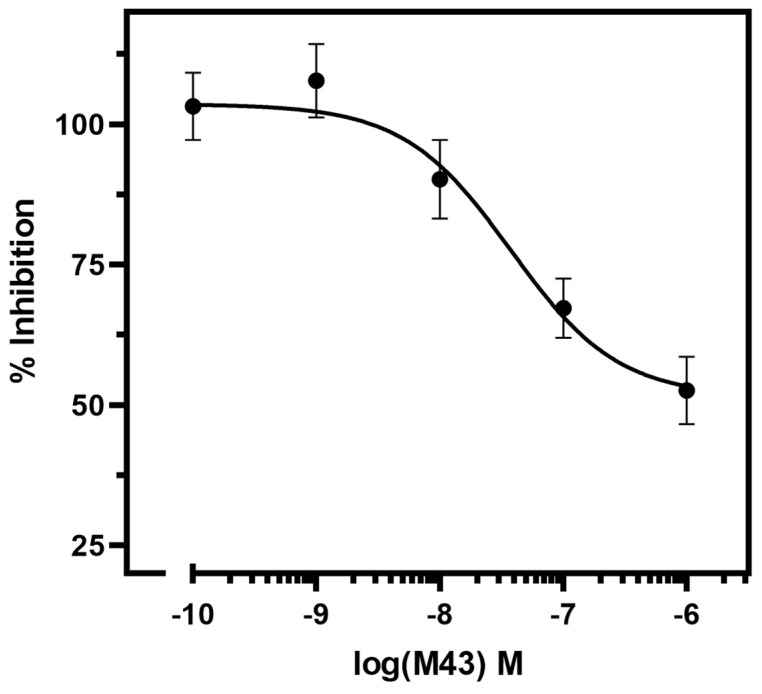
M43 inhibits cAMP accumulation stimulated by 10 nM of Tyr^0^-sauvagine. The stimulation of cAMP accumulation in HEK 293 cells stably expressing the CRF_1_R by 10 nM of Tyr^0^-sauvagine in the absence or presence of increasing concentrations of M43 was determined as described in the “Materials and Methods”. Data represent the mean ± S.E. from six independent experiments. The cAMP accumulation was normalized by assigning 100% to the maximal response after stimulation with Tyr^0^-sauvagine in the absence of M43.

To further examine the antagonistic properties of M43, we determined its half-maximal inhibitory concentration (or antagonistic potency, pIC_50_). The IC_50_ of M43 is determined as its concentration required to decrease the cAMP accumulation stimulated by 10 nM Tyr^0^-sauvagine to half. As shown in Figure 6, M43 inhibited cAMP accumulation stimulated by 10 nM Tyr^0^-sauvagine in a dose–response manner, with an antagonistic potency of 43.5 nM (pIC_50_ = 7.36 ± 0.18, n = 6).

### 3.4. M43 Antagonizes the Effect of Tyr^0^-Sauvagine on the Proliferation of RAW 264.7 Macrophages

**Figure 7:**
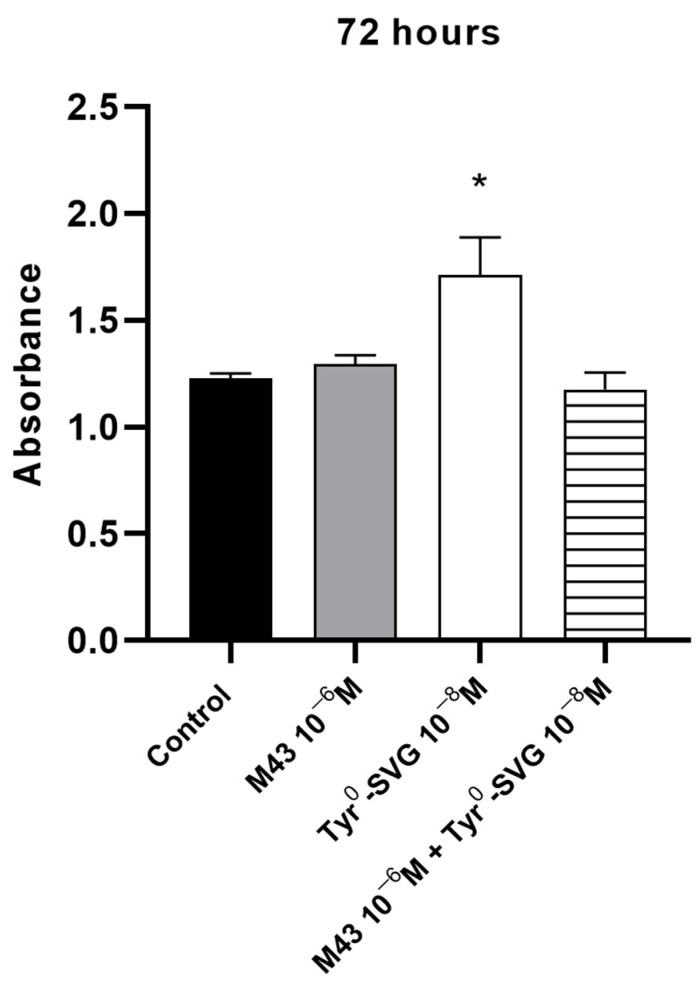
M43 inhibits the proliferation rate of RAW 264.7 macrophages stimulated by 10 nM of Tyr^0^-sauvagine. The proliferation rate of RAW 264.7 macrophages following 72 h treatment with 10 nM of Tyr^0^-sauvagine in the absence or presence of 1 μΜ of M43 was determined as described in the “Materials and Methods”. The means and S.E. are shown from a representative experiment. (*) Represents a comparison between the 10 nM Tyr^0^-sauvagine group and 10 nM Tyr^0^-sauvagine+1 μΜ M43 group, n = 5. * *p* < 0.01. Statistical analysis was performed using one-way ANOVA, followed by Tukey’s multiple comparison test.

To support the antagonistic properties of M43, we determined whether this molecule could block the effects of Tyr^0^-sauvagine to induce the proliferation of RAW 264.7 macrophage cells. Cells were incubated with 10 nM of Tyr^0^-sauvagine in the presence or absence of (control) of 1 μΜ of M43. As shown in Figure 7, the stimulation of the proliferation rate of RAW 264.7 cells by 10 nM of Tyr^0^-sauvagine after 72 h was significantly inhibited by M43 at a concentration of 1 μΜ.

## 4. Discussion

In our previous study, we have designed, synthesized, and tested the abilities of 45 novel non-peptide molecules to bind to CRF_1_R [33]. Among these compounds, the analogs M6, M7, M8, M21, M22, M31, M42, and M43 bound to CRF_1_R with high affinities (41, 160.7, 94.6, 173.4, 276.7, 66.2, 145.2, and 19.2 nM, respectively), with M43 being the best binder (Figure 1). Molecular modeling studies performed in the present study highlighted the essential role of the hydrogen bond between N283^5.50b^ of CRF_1_R and the common pyridine nitrogen of analogs M6, M7, M8, M21, M22, M31, M42, and M43, as well as all other compounds tested in our previous study [33]. In addition, the three chlorides of the phenyl group that exist in several compounds seem to have equally distributed charges, facilitating hydrophobic interactions, compared to the bromide and isopropyl groups of other compounds, thereby yielding better docking results. Comparison of the analogs M6 and M8, which have similar chemical structures but different groups at the tertiary amine position, suggests that the presence of ethyl and butyl groups (M6) at this position contributes to a higher affinity binding than the presence of two ethyl groups (M8), perhaps due to the tunnel formed toward the membrane (Figure 2). A similar relationship exists between the chemical groups at position 7 and the binding affinity of the analogs M43 (with ethyl and butyl groups) and M42 (with two ethyl groups). Molecular modeling data suggest that the contribution of the butyl group at the tertiary amine position of molecules to their high-affinity binding is mostly due to its interaction with L320^6.46b^ of CRF_1_R and the membrane. A comparison of M43 with other compounds featuring ethyl and butyl groups at the tertiary amine position, including M6, and analysis of molecular dynamics simulations suggest that the presence of the oxime hydroxyl group in M43 could explain its higher binding affinity compared with the other molecules. This hydroxyl group of M43 possibly forms an extra hydrogen bond with the main chain carbonyl oxygen or the side chain hydroxyl of Thr316^6.42b^ (Figure 4).

Non-peptide CRF_1_R antagonists—including antalarmin and more clinically advanced scaffolds, such as tildacerfont and crinecerfont—bind the intramembrane, allosteric site that is buried within the transmembrane (TM) bundle and distinct from the N-terminal peptide orthosteric site. This site is primarily formed by TM3, TM5, and TM6, and antagonist binding stabilizes inactive-state conformations that impede the outward displacement of TM6 required for G_s_ engagement and signaling. Our AlphaFold3 models and MD simulations place M43 in this canonical allosteric cleft and recapitulate the class-defining anchors: a persistent H-bond to N283^5.50b^ and a complementary, switchable H-bonding interaction with T316^6.42b^ (backbone carbonyl vs. side-chain hydroxyl). In contrast, the *N*-ethyl–*N*-butyl side chain extends toward a hydrophobic, membrane-facing subpocket (Figure 2 and Figure 4). This pose and network are fully consistent with the allosteric antagonism established for CRF_1_R [34,36,42]. Mechanistically, CRF_1_R small-molecule antagonists commonly display insurmountable antagonism attributed to slow dissociation from the TM pocket; the persistent inhibition we observe for M43 is consonant with this kinetic trapping paradigm [21,42].

The ability of M43 to bind to CRF_1_R with the highest binding affinity compared to the other tested compounds prompted us to further pharmacologically characterize it by determining its ability to antagonize the CRF-related agonist, Tyr^0^-sauvagine. M43 is a CRF potent antagonist, blocking the ability of 10 nM Tyr^0^-sauvagine to stimulate cAMP accumulation in HEK 293 cells that stably express the CRF_1_R, displaying a half-maximal inhibitory concentration of 43.5 nM. As a potent antagonist, M43, at a concentration of 1 μM, was able to reduce the potency of Tyr^0^-sauvagine to stimulate the intracellular cAMP accumulation by 6.2-fold. Similarly, the prototype CRF_1_R selective antagonist antalarmin decreased the potency of Tyr^0^-sauvagine by 33-fold to stimulate cAMP accumulation in HEK 293 expressing the CRF_1_R [34]. The lower antagonistic ability of M43 compared to that of antalarmin could be attributed to its lower binding affinity (19.2 nM) compared with antalarmin (9.7 nM) [33]. To provide supportive evidence for the antagonistic properties of M43, we tested its ability to block the effect of Tyr^0^-sauvagine on the proliferation of the RAW 264.7 macrophage cells [44]. Our data suggested that M43 antagonized Tyr^0^-sauvagine, thereby stimulating the proliferation of RAW 264.7 cells.

The activation of CRF_1_R is associated with the disruption of interactions between receptor amino acids, and the formation of a new comparison of the high-resolution inactive and active structures of CRF_1_R suggests that its activation is closely related to structural changes, involving an upward movement of the fourth (TM4) and fifth (TM5) transmembrane domains that repositions the second extracellular (EL2) and intracellular (IL2) loops [45]. Receptor activation is also associated with other orchestrated movements of the transmembrane domains, including the third (TM3), fifth, and sixth (TM6) ones [34,45]. These movements are responsible for the high-affinity binding of agonists to the receptor and the interaction of the latter with the G-proteins. M43 most likely impedes the activation-associated movements of CRF_1_R by interacting with the described allosteric pocket formed by TM3, TM5, and TM6, as shown in Figure 4. This conformational trapping provides a coherent structural basis for the insurmountable-like antagonism observed across this class of molecules [21,42]. While our modeling and cellular pharmacology place M43 firmly within the CRF_1_R allosteric antagonist class and align its behavior with antalarmin and clinically advanced scaffolds, additional experiments would sharpen differentiation. Such data will enable a more granular, mechanism-focused comparison of M43 with antalarmin, tildacerfont, and crinecerfont.

In future studies, we will evaluate the in vivo pharmacological properties and toxicities of the thiazolopyrimidine analog M43, as well as its ability to act on different GPCRs such as serotonin or adenosine receptors, which play crucial roles in depression [14,46,47]. Previous studies have shown that thiazolopyrimidine derivatives are antagonists for serotonin and adenosine receptors [48,49]. Evaluating the thiazolopyrimidine derivative M43 against other GPCRs could reveal an important pharmacological property, namely, a desirable non-selectivity for different GPCRs. As proposed by Roth et al., non-selective molecules could be used as “magic shotguns” to treat CNS disorders, including depression [50].

## 5. Conclusions

The results of this study suggest that the analog M43 is a potent CRF_1_R antagonist that could be used as a lead molecule for the development of novel non-peptide CRF_1_R antagonists. The antagonistic properties of M43 are attributed to its interaction with residues in the TM3, TM5, and TM6 of CRF_1_R. These interactions block activation-associated structural rearrangements of the CRF_1_R. The pharmacological properties of M43 render it an optimal lead compound in the rational design of novel non-peptide CRF antagonists, which will enrich the pharmaceutical arsenal against CAH and stress-related disorders.

## Data Availability

Data are contained within the article.

## Supplementary Materials

The following supporting information can be downloaded at https://www.mdpi.com/article/10.3390/biom15091265/s1: Table S1: Codes and chemical names of the compounds in this study. Figure S1: AphaFold3 models of CRF_1_R in a complex with M43 and antalarmin, as well as a comparison with the crystal structure of CRF_1_R with BMK-C205; Figure S2: Three-dimensional representation of residue flexibility during the simulation; Figure S3: Model comparison with the MD representative state and the crystal structure complex of the receptor bound to BMK-C205, as well as RMSD calculations compared to the BMK-C205-CRF_1_R structure.

## Abbreviations

The following abbreviations are used in this manuscript:

CRF	corticotropin-releasing factor
CRF1R	type 1 receptor for the corticotropin-releasing factor
HPA	hypothalamic–pituitary–adrenal
CAH	congenital adrenal hyperplasia
ACTH	adrenocorticotropic hormone or corticotropin
HEK	human embryonic kidney
DMEM	Dulbecco’s modified Eagle’s medium
cAMP	cyclic adenosine monophosphate
MTT	(3-(4,5-dimethylthiazol-2-yl)-2,5-diphenyltetrazolium bromide
pLDDT	predicted local distance difference test
PAE	predicted alignment error
POPC	1-palmitoyl-2-oleoyl-sn-glycero-3-phosphocholine
CGenFF	CHARMM General Force Field
ECD	extracellular domain
TCA	trichloroacetic acid
PME	Particle-Mesh Ewald
