# Supplementary material for: Pharmacological Characterization of the Novel CRF1 Receptor Antagonist, Thiazolo[4,5-d] Pyrimidine Analog, M43
Source: Biomolecules. 2025 Sep 1;15(9):1265. doi: 10.3390/biom15091265 (PMC12467512; doi:10.3390/biom15091265)
Supplement: Supplementary file 1 [file biomolecules-15-01265-s001.zip › biomolecules-3798308-supplementary.pdf]

SUPPLEMENTARY MATERIALS

Pharmacological characterization of the novel CRF1 receptor antagonist, thiazolo[4,5-d]pyrimidine analog, M43

Spyridon Marios Giatro[1#], George Komontachakis[2#], Aikaterini Kalantidou[3], Nastazia Lesgidou[1], Vlasios Karageorgos[2], Mohamed Teleb[4,5], Md Rabiul Islam[6], Thomas Mavromoustakos[7], Hesham Fahmy[6], Maria Venihaki[3], Minos-Timotheos Matsoukas[1]* and George Liapakis[2]*

1 Department of Biomedical Engineering, University of West Attica, Egaleo 12210, Athens, Greece; sgiatro@uniwa.gr (S.M.G.); nlesgidou@uniwa.gr (N.L.); mmatsoukas@uniwa.gr (M.-T.M.).

2 Department of Pharmacology, School of Medicine, University of Crete, Heraklion, 71003, Crete, Greece; geokomontas@gmail.com (G.K.); bkarageorgos@hotmail.com (V.K.); liapakig@uoc.gr (G.L.)

3 Department of Clinical Chemistry, School of Medicine, University of Crete, Heraklion, 71003, Crete, Greece; katekld7@gmail.com (A.K.); venycham@uoc.gr (M.V.)

4 Department of Pharmaceutical Chemistry, Faculty of Pharmacy, Alexandria University, Alexandria, 21521, Egypt

5. Department of Medicinal Chemistry, Faculty of Pharmacy, Alamein International University, Alamein 51718, Egypt

6 Department of Pharmaceutical Science, College of Pharmacy & Allied Health Professions, South Dakota State University, Brookings, SD, 57007, USA; mdrabiul.islam925@jacks.sdstate.edu (M.R.I.); hesham.fahmy@sdstate.edu (H.F.)

7 Department of Chemistry, National and Kapodistrian University of Athens, Panepistimiopolis Zografou 15771, Greece; tmavrom@chem.uoa.gr (T.M.)

* Correspondence: liapakig@uoc.gr (G.L.); mmatsoukas@uniwa.gr (M.-T.M.)

\# These authors contributed equally to this work

Table S1. Codes and chemical names of the compounds in this study.

Code	Chemical Name	Functional Groups
M6	7-(N-butyl-N-ethylamino)-3-(2-bromo-4-isopropylphenyl)-5-methyl-2-(methylimino)-2,3-dihydrothiazolo[4,5-d]pyrimidine	Thiazolopyrimidine fused N,S-heterobicycle; tertiary dialkylamine (N-ethyl-N-butyl); exocyclic imine (C=N–Me); bromide; isopropyl-phenyl; methyl.
M7	7-(N-butyl-N-ethylamino)-3-(2-bromo-4-isopropylphenyl)-5-methyl-2-(propylimino)-2,3-dihydrothiazolo[4,5-d]pyrimidine	Thiazolopyrimidine fused N,S-heterobicycle; tertiary dialkylamine; exocyclic imine (C=N–Pr); aryl bromide; isopropyl-phenyl; methyl.
M8	7-(diethylamino)-3-(2-bromo-4-isopropylphenyl)-5-methyl-2-(methylimino)-2,3-dihydrothiazolo[4,5-d]pyrimidine	Thiazolopyrimidine fused N,S-heterobicycle; tertiary dialkylamine (diethylamino); exocyclic imine (C=N–Me); aryl bromide; isopropyl-phenyl; methyl.
M21	(N-butyl-N-ethylamino)-3-(2-bromo-4-isopropylphenyl)-5-methyl-2-imino-2,3-dihydrothiazolo[4,5-d]pyrimidine	Thiazolopyrimidine fused N,S-heterobicycle; tertiary dialkylamine; exocyclic imine (C=NH); aryl bromide; isopropyl-phenyl; methyl.
M22	7-(diethylamino)-3-(2-bromo-4-isopropylphenyl)-5-methyl-2-imino-2,3-dihydrothiazolo[4,5-d]pyrimidine	Thiazolopyrimidine fused N,S-heterobicycle; tertiary dialkylamine (diethylamino); exocyclic imine (C=NH); aryl bromide; isopropyl-phenyl; methyl.
M31	7-(Butyl,ethyl)amino-5-methyl-3-(2,4,6-trichlorophenyl)thiazolo[4,5-d]pyrimidin-2(3H)-methylimine	Thiazolopyrimidine fused N,S-heterobicycle; tertiary dialkylamine; exocyclic imine (C=N–Me); polychlorinated aryl (2,4,6-trichlorophenyl); methyl.
M42	7-(diethylamino)-2-(hydroxyimino)-5-methyl-3-(2,4,6-trichlorophenyl)-2,3-dihydrothiazolo[4,5-d]pyrimidine	Thiazolopyrimidine fused N,S-heterobicycle; tertiary dialkylamine (diethylamino); oxime; polychlorinated aryl; methyl.
M43	7-(N-butyl-N-ethylamino)-2-(hydroxyimino)-5-methyl-3-(2,4,6-trichlorophenyl)-2,3-dihydrothiazolo[4,5-d]pyrimidine	Thiazolopyrimidine fused N,S-heterobicycle; tertiary dialkylamine; oxime; polychlorinated aryl aryl (2,4,6-trichlorophenyl); methyl.

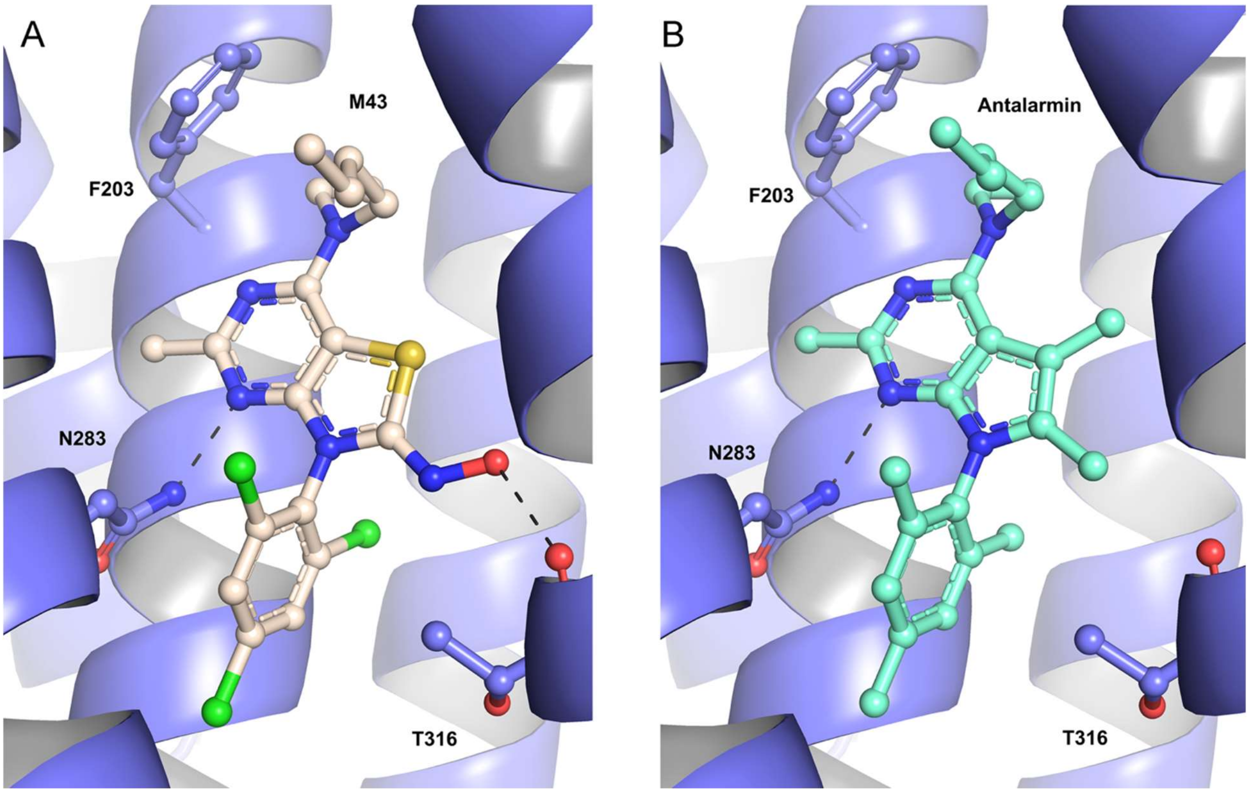

Figure S1. Aphafold3 models of CRF_1R in complex with A) M43 and B) Antalarmin.

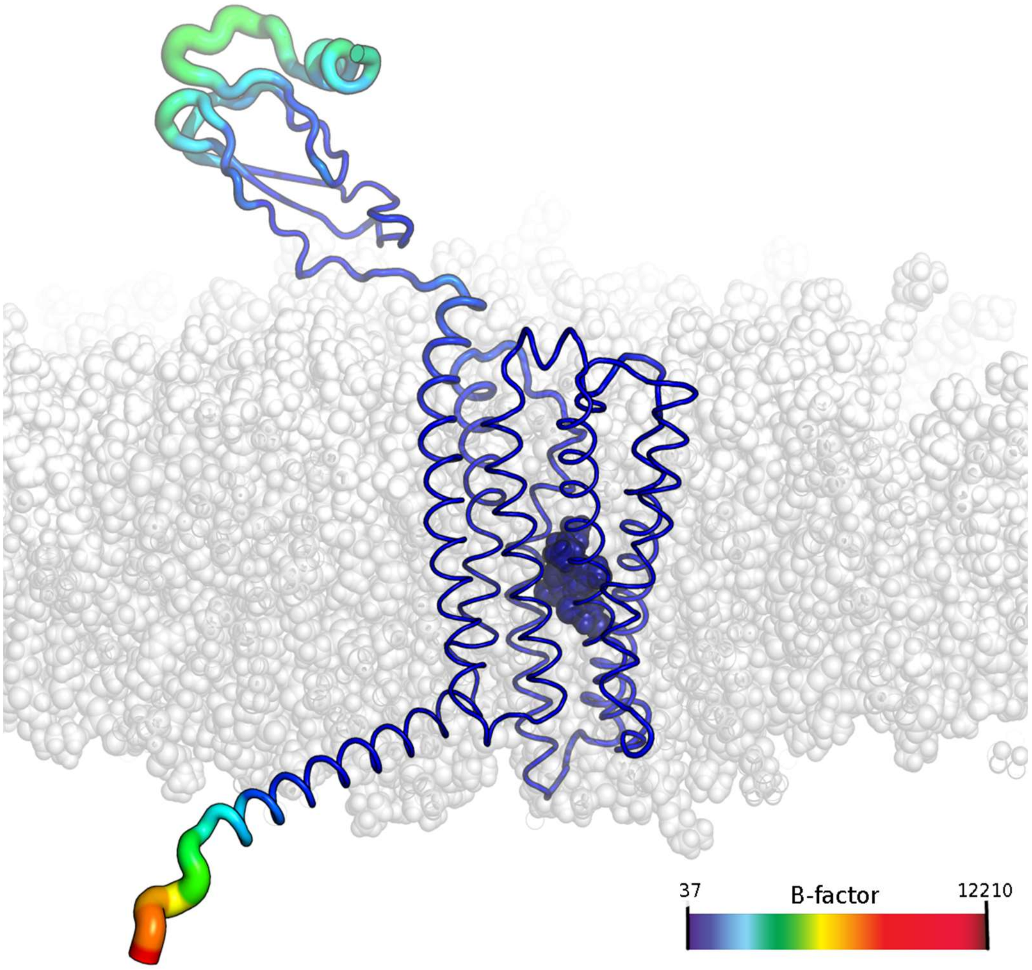

Figure S2. 3D representation of residue flexibility during the simulation. The protein is shown in putty cartoon style, with both color and tube thickness representing B-factors calculated from the trajectory. Thicker and more intensely colored regions correspond to higher atomic fluctuations. The B-factor values range from 37 to 12210.

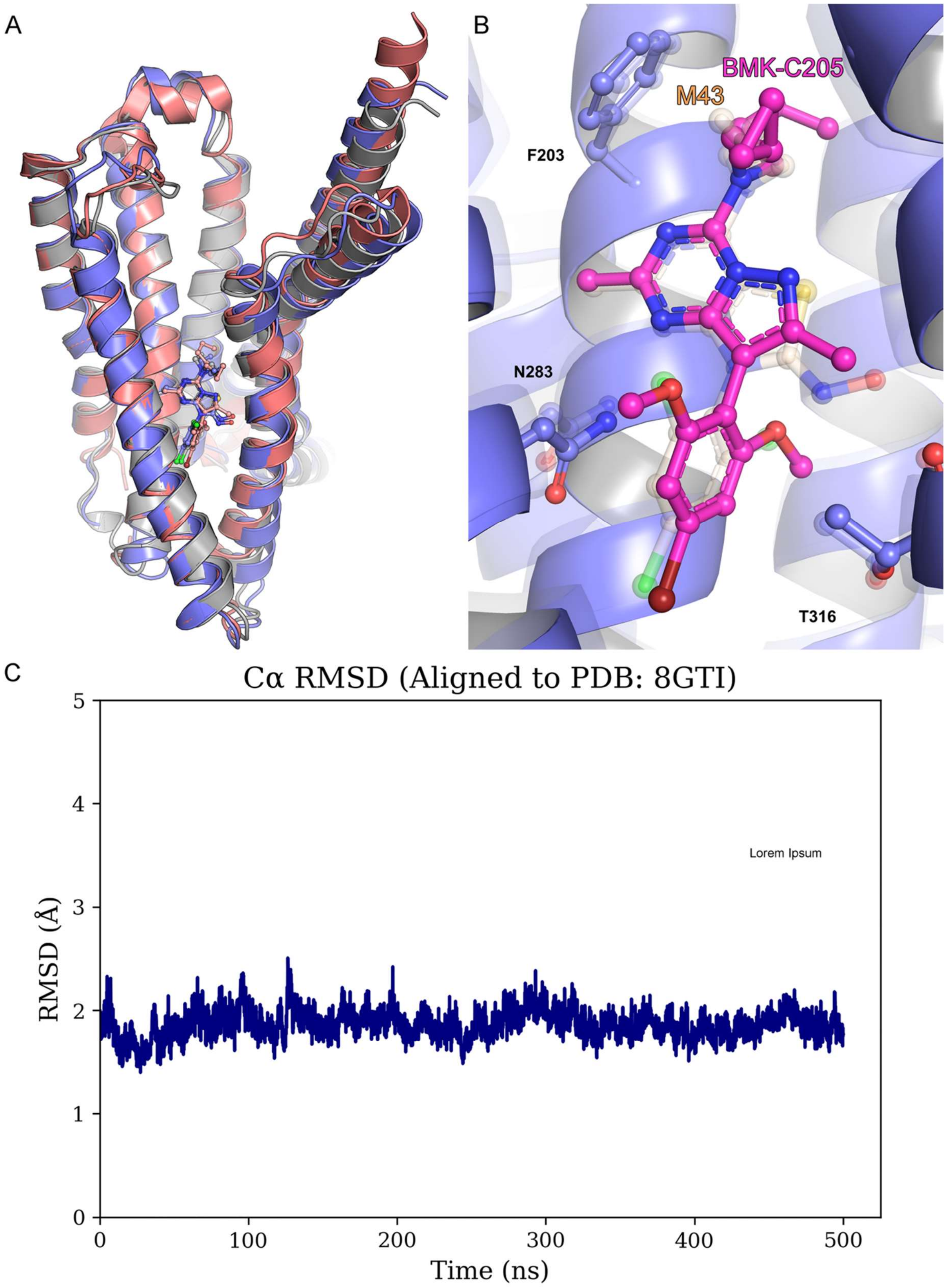

Figure S3. A. Close view of molecules BMK-C205 (magenta) (pdb: 8gti) and M43 (transparent orange) from crystal structure and Alphafold model, respectively. B. Superimposition of the Alphafold model used in this study (grey), the most representative state from the MD simulation at 251500ps (blue) and the complex of the receptor bound to BMK-C205 (pdb: 8gti) crystal structure (pink). C. RMSD calculation (only TMD) of frames from the M43-CRF_1R complex simulation, with the BMK-C205-CRF_1R structure.
